# Comprehensive analysis of NT5DC family prognostic and immune significance in breast cancer

**DOI:** 10.1097/MD.0000000000032927

**Published:** 2023-02-10

**Authors:** Yiwei Jia, Jia Li, Huizi Wu, Weiwei Wang, Shiyu Sun, Cong Feng, Xuan Liu, Chaofan Li, Yu Zhang, Yifan Cai, Xinyu Wei, Peizhuo Yao, Xuanyu Liu, Shuqun Zhang, Fei Wu

**Affiliations:** a Collage of Clinical Medicine, Xi’an Jiaotong University, Xi’an, PR China; b Department of Oncology, The Second Affiliated Hospital of Xi’an Jiaotong University, Xi’an, PR China.

**Keywords:** biomarkers, breast cancer, immune, prognosis, NT5DC family

## Abstract

Among the most common malignancies, breast cancer has a high incidence and mortality rate. NT5DC family is a highly well-conserved 5′-nucleotidase. Previous studies showed that the progression of tumors was associated with some NT5DC family members. However, there are no studies about the comprehensive analysis such as expression, prognosis, and immune properties of NT5DC family in breast cancer. Based on the data from The Cancer Genome Atlas database, we used UALCAN, Tumor Immune Estimation Resource, Breast cancer gene-expression miner (Bc-GenExMiner), Kaplan–Meier Plotter, TISIDB, cBioPortal, GeneMANIA, Search Tool for the Retrieval of Interacting Genes, Metascape, Tumor Immune Single-cell Hub, The Database for Annotation, Visualization and Integrated Discovery, and Gene Set Cancer Analysis databases to explore expression, prognostic and diagnostic value, genetic alterations, biological function, immune value and drug sensitivity of NT5DC family in breast cancer patients. There was a downregulation of NT5C2, NT5DC1, and NT5DC3 in breast cancer compared to normal tissues, and NT5DC2 instead. All NT5DC family members were associated with the clinicopathological parameters of breast cancer patients. Survival and ROC analysis revealed that NT5DC family genes were related to the prognosis and diagnosis of breast cancer. NT5DC family were mainly involved in nucleotide metabolism. Moreover, NT5DC family were significantly associated with tumor immune microenvironment, diverse immune cells, and immune checkpoints in breast cancer. This research showed that NT5DC family might be novel prognostic biomarkers and immunotherapeutic targets of breast cancer.

## 1. Introduction

Breast cancer is now the fifth most common cause of cancer-related mortality (6.9% of all cases), and the most commonly diagnosed malignant cancer (11.7% of all cases) worldwide. The American Cancer Society reports that in 2020 there were nearly 2,261,419 new cases of breast cancer and 684,996 deaths.^[1]^ Breast cancer is classified into four subtypes based on the expression of estrogen receptor, progesterone receptor, and human epidermal growth factor receptor 2 (HER2), including basal-like, luminal A, luminal B, and HER2 enriched molecular subtypes.^[2]^ In the past few years, numerous advances have been made in the diagnosis and treatment of cancer. The use of biomarkers, such as estrogen receptor, progesterone receptor, and HER-2, for predicting breast cancer outcome or targeting endocrine therapy is widespread.^[3]^ The diagnosis and treatment of breast cancer has progressed over the past few decades, however, recurrence and metastasis is still incurable and has become the biggest challenge in clinical treatment.^[4]^ Therefore, finding new biomarkers and discovering their mechanisms in breast cancer is crucial for the diagnosis, treatment, and prognosis of patients with breast cancer.

NT5DC (5′-Nucleotidase domain containing) family is an extremely well-conserved 5′-nucleotidase, which have a haloacid dehalogenase motif in their N-termini, including NT5C2, NT5DC1, NT5DC2, NT5DC3, and NT5DC4.^[5]^ NT5DC family members participated in hereditary spastic paraplegia, mental disorders, and metabolic disorders.^[6]^ Recently, multiple studies have linked NT5DC family members to tumor progression. In acute myeloid leukemia patients, higher expression of NT5C2 related to poor prognostic.^[7–9]^ In lung tumor cells, the silence of NT5C2 opposes the cancer phenotype, activates the p53/AMPK pathway and regulates metabolism.^[10,11]^ Raza et al^[12]^ showed that NT5C2 deficiency facilitates tumor growth, response to chemotherapeutic and migration. When treating non-small cell lung cancer cells with gemcitabine and platinum, the expression of NT5C2 can be used as an indicator of response.^[13,14]^ In hepatocellular carcinoma, NT5DC2 increases tumor cell proliferation by upregulating EGFR expression^[15]^ and can be used as a prognostic biomarker.^[16]^ Zhu et al^[17]^ revealed that colorectal carcinoma cells were reduced when NT5DC2 expression was suppressed, resulting in a reduction in proliferation, migration, invasion and infiltration of tumor-associated macrophages. Li et al^[18]^ showed that hepatocellular carcinoma expression of the NT5DC gene family was related to prognosis and immune infiltration. In breast cancer, only one study suggested that NT5DC2 take part in glucose deprivation through ROS metabolism and defense.^[19]^ The role of NT5DC family in breast cancer is unclear, and there are no studies about the expression, prognosis, and immune properties of NT5DC family.

This study explored the expression, the diagnostic and prognostic value of NT5DC family for patients with breast cancer by integrating various bioinformatics methods. Furthermore, we revealed the close correlation between NT5DC family and immune infiltration.

## 2. Materials and Methods

### 2.1. The Cancer Genome Atlas (TCGA)

We obtained the RNAseq data (level 3 HTSeq-FPKM) of breast cancer from TCGA database (https://portal.gdc.cancer.gov/), including 1109 tumor samples and 113 normal samples. RNAseq data in FPKM (Fregments Per Kilobase per Million) format was converted into TPM (transcripts per million reads) format and log2 conversion was performed.

### 2.2. UALCAN

Data from the UALCAN database was used to determine the expression of the NT5DC family in breast cancer (http://ualcan.path.uab.edu/). UALCAN is a web-portal that allows users to analyze gene expression data from the TCGA.^[20]^ Additionally, NT5DC expression was correlated with tumor stage using this method.

### 2.3. Tumor Immune Estimation Resource (TIMER)

TIMER database(http://timer.cistrome.org/) is a web-based tool that lets users explore the relationships between immune infiltrates and a wide-spectrum of factors.^[21]^ NT5DC family expression was compared between breast cancer and adjacent normal tissues using the DiffExp module. Gene module analysis correlated NT5DC family members with immune cell infiltration in breast cancer. Moreover, NT5DC family genetic copy number changes were associated with the abundance of immune infiltrates in the SCNA module, which was applied to the analysis.

### 2.4. Breast Cancer Gene-Expression Miner (Bc-GenExMiner)

Our study utilized the Bc-GenExMiner v4.8(http://bcgenex.ico.unicancer.fr/BC-GEM/GEM-Accueil.php) to examine the relationship between the expression levels of NT5DC family and Scarff-Bloom-Richardson (SBR) grade and Nottingham Prognostic Index (NPI). Survival analysis was also exerted with it. It is an easy-to-use database to let users mine published data of breast cancer.^[22]^

### 2.5. Kaplan–Meier Plotter

To assess the prognostic value, mRNA expression levels of the NT5DC family were analyzed using Kaplan–Meier Plotter (http://kmplot.com/analysis/), which can perform Cox regression and construct Kaplan–Meier plots.^[23]^ The overall survival (OS), relapse-free survival (RFS), distant metastasis-free survival (DMFS), and post progression survival (PPS) between the two groups of patients was evaluated after dividing the patients into two groups (high vs. low expression).

### 2.6. TISIDB

TISIDB (http://cis.hku.hk/TISIDB) integrates many data sources from oncoimmunology to enable thorough research on tumor–immune interactions.^[24]^ It was used to investigate the relationship between the expression of the NT5DC family members and the immunological subtypes of breast cancer. The relations between NT5DC family and immunoinhibitor or immunostimulator were also analyzed with it.

### 2.7. cBioPortal

The cBioPortal database (http://cbioportal.org) can explore, visualize, and interpret cancer genomics data.^[25]^ Genetic alternation and DNA methylation of NT5DC family members in breast cancer were obtained via cBioPortal.

### 2.8. GeneMANIA

A flexible web tool called GeneMANIA (http://www.genemania.org) is used to prioritize genes for functional experiments, analyze gene lists, and create hypotheses about the function of genes.^[26]^ We used it to explore the relationship between NT5DC family and their related genes.

### 2.9. Search Tool for the Retrieval of Interacting Genes (STRING)

The STRING database(https://string-db.org/) is an online website that strives to compile, evaluate, and integrate all publicly accessible sources of data about protein-protein interactions.^[27]^ In the study, the PPI network of NT5DC family was elucidata with STRING.

### 2.10. Metascape

Metascape (https://metascape.org/gp/) is an efficient tool to comprehensively interpret OMICs-based studies.^[28]^ Herein, we employed this database to perform enrichment analysis on NT5DC family related genes.

### 2.11. The Database for Annotation, Visualization and Integrated Discovery (DAVID)

A comprehensive set of tools is provided by DAVID (https://david.ncifcrf.gov/) that allow researchers to make sense of large lists of genes and their biological functions.^[29]^ NT5DC family and close related neighbor genes were analyzed by gene ontology (GO) enrichment and Kyoto Encyclopedia of Genes and Genomes (KEGG) pathway enrichment analysis using DAVID database and visualized with R project using a “ggplot2” package and *P* < .05.

### 2.12. Tumor Immune Single-cell Hub (TISCH)

An easy-to-use interface is provided by the TISCH database (http://tisch.comp-genomics.org/home/) to systematically visualize, search, and download gene expression atlases in tumor microenvironment.^[30]^ We used it to evaluate the tumor microenvironment in three datasets.

### 2.13. Single sample Gene Set Enrichment Analysis (ssGSEA)

Using the GSVA package (http://www.bioconductor.org/packages/GSVA/) in R, we estimated the associations of twenty-four types of immune-related cells and NT5DC family members through the ssGSEA algorithm.

### 2.14. Estimation of Stromal and Immune cells in Malignant Tumor tissues using Expression data (ESTIMATE)

An algorithm for predicting tumor purity in tumor immune environment, ESTIMATE, included stromal score, immune score, and estimate scores.^[31]^ We used ESTIMATE package (https://sourceforge.net/projects/estimateproject/) in R to observe the relationship between NT5DC family expression and tumor immune microenvironment in breast cancer.

### 2.15. Gene Set Cancer Analysis (GSCALite)

GSCALite (http://bioinfo.life.hust.edu.cn/web/GSCALite/) is an online website which uses big multi-omics and drug data to provide comprehensive analysis for a set of genes in cancers.^[32]^ The drug sensitivity analysis was conducted based on the Genomics of Drug Sensitivity in Cancer (GDSC) and Cancer Therapeutics Response Portal (CTRP) databases through it.

### 2.16. Statistical analysis

Bioinformatic statistics analyses were carried out using R v 4.1.1. Receiver operating characteristic (ROC) analysis was conducted to evaluate the diagnostic value of NT5DC family using the “pROC” package of R. *t* test was employed to evaluate NT5DC family expression in multiple tissues. Using the log-rank test, survival analysis was performed. The Spearman analysis was conducted to calculate the correlation coefficients. *P* < .05 was considered statistically significant.

### 2.17. Ethical statements

As all data were obtained from public database, this study did not require ethical approval.

## 3. Results

### 3.1. Expression of NT5DC family in breast cancer patients

**Figure 1.**
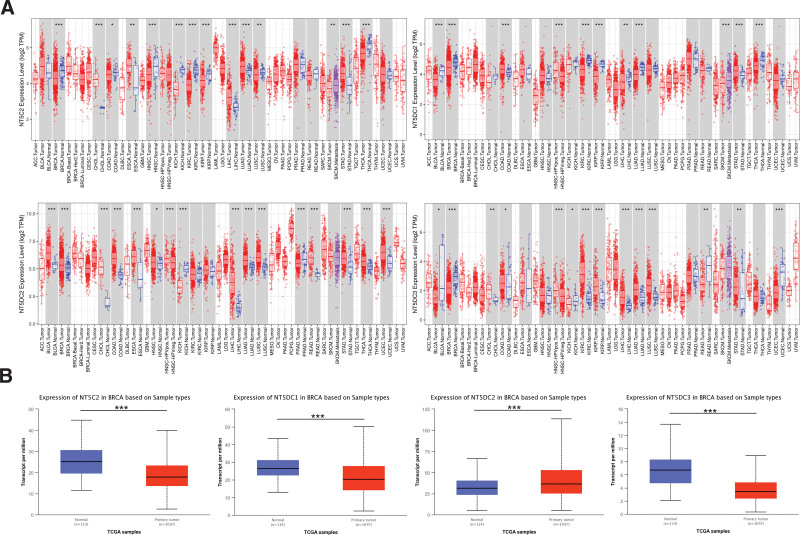
The expression levels of the NT5DC family in breast cancer. (A) The mRNA expression of NT5DC family in the TIMER database. (B) The mRNA expression of NT5DC family in the UALCAN database. *P < .05; **P < .01; ***P < .001; BRCA = breast invasive carcinoma, NT5DC = 5′-Nucleotidase domain containing, TCGA = The Cancer Genome Atlas, TIMER = Tumor Immune Estimation Resource.

We explore the expression of the NT5DC family in breast cancer patients in TIMER and UALCAN databases. The results of the TIMER database showed that the expression of NT5C2, NT5DC1, and NT5DC3 were significantly downregulated in breast cancer tissues, while the expression of NT5DC2 were upregulated. At the same time, we compared the expression of NT5DC family in breast cancer and normal breast tissues in the UALCAN database (Fig. 1B), and the results were the same as the TIMER database. The expression of NT5DC4 was not explored in these two databases.

### 3.2. Association between NT5DC family expression and the clinicopathological parameters of breast cancer patients

**Figure 2.**
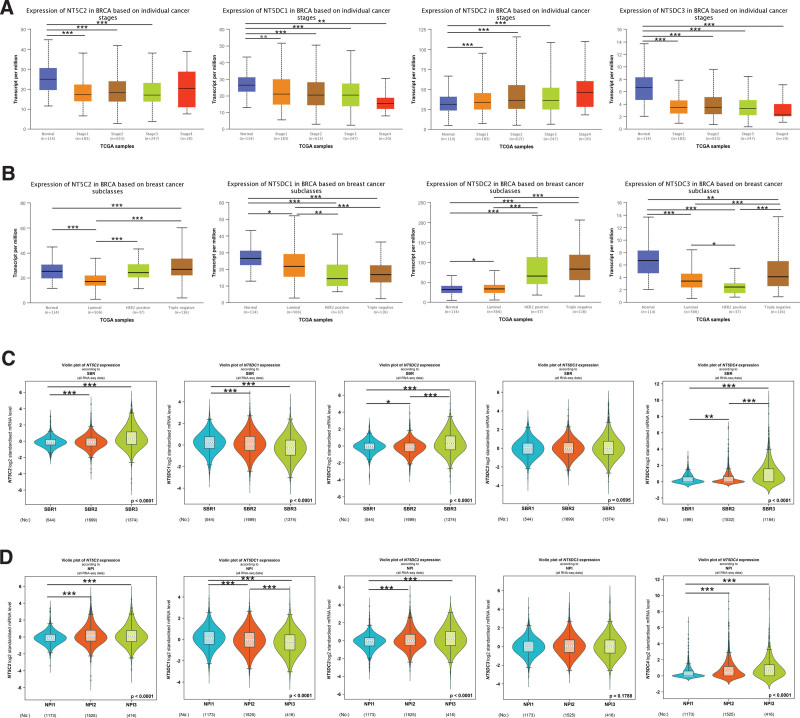
Clinicopathological parameters associated with NT5DC family mRNA expression in breast cancer. (A) Boxplots showed NT5DC family transcript levels based on breast cancer stage in the UALCAN database. (B) Boxplots showed NT5DC family expression in breast cancer based on molecular subtypes in the UALCAN database. (C) Violin plots showed NT5DC family expression in breast cancer according to SBR in the Bc-GenExMiner database. (D) Violin plots showed NT5DC family expression in breast cancer based on NPI. *, *P* < .05; **, *P* < .01; ***, *P* < .001; BRCA = breast invasive carcinoma, NPI = Nottingham Prognostic Index, NT5DC = 5′-Nucleotidase domain containing, SBR = Scarff-Bloom-Richardson, TCGA = The Cancer Genome Atlas.

We then assessed the correlation between the expression of the NT5DC family and the clinicopathological parameters of breast cancer patients through UALCAN and Bc-GenExMiner databases. Compared with normal tissues, the expression levels of NT5C2, NT5DC1 and NT5DC3 in stage1 to 4 were significantly reduced, while the expression level of NT5DC2 was significantly increased (Fig. 2A). Regarding the molecular subtypes of breast cancer (Fig. 2B), NT5C2 expression was significantly increased in patients with triple-negative breast cancer (TNBC) subtype, and reduced in patients with Luminal subtype. NT5DC1 and NT5DC3 expression were strongly reduced in Luminal, HER2 Positive and TNBC subtypes. NT5DC2 expression. NT5DC2 expression was significantly increased in patients with Luminal, HER2 Positive and TNBC subtypes. In relation to the prognostic factors in breast cancer (Figs. 2C and D), higher expression of NT5C2, NT5DC2 and NT5DC4 had more advanced SBR grades and higher NPI values; by contrast, lower expression of NT5DC1 had more advanced SBR grades and higher NPI values. According to these results, the NT5DC family mRNA expression was significantly correlated with clinicopathological parameters.

### 3.3. Prognostic and diagnostic role of NT5DC family in breast cancer patients

**Figure 3.**
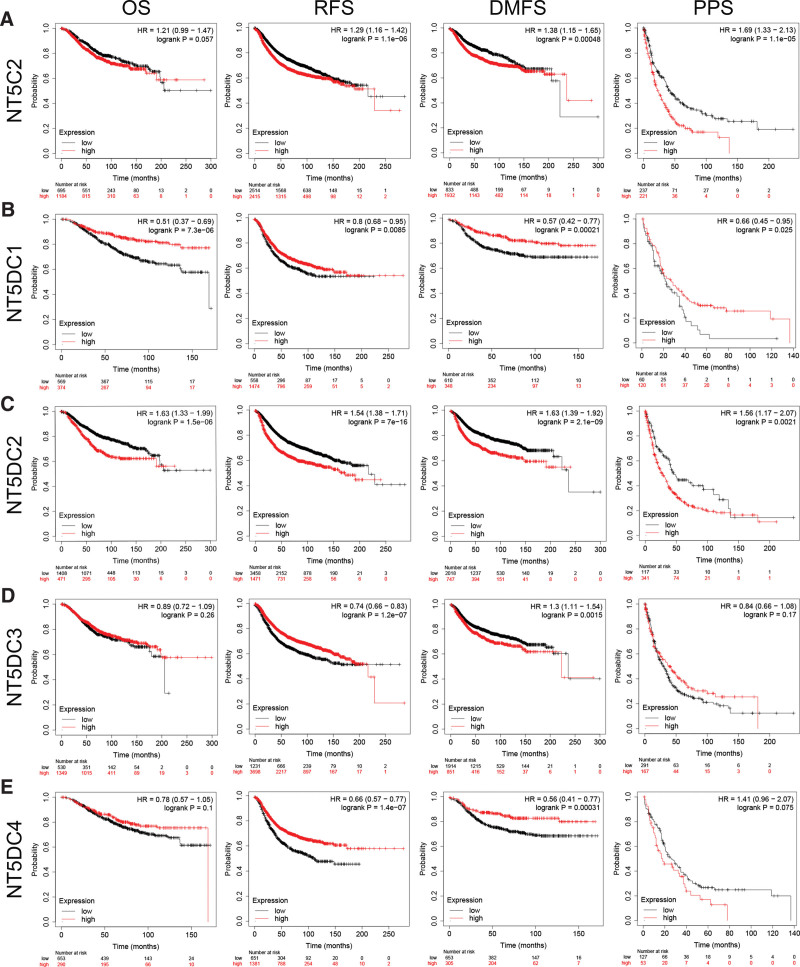
The prognostic value of NT5DC family in breast cancer via Kaplan–Meier Plotter. The survival plots of NT5C2 (A), NT5DC1 (B), NT5DC2 (C), NT5DC3 (D), and NT5DC4 (E). DMFS = distant metastasis-free survival, HR = hazard ratio, NT5DC = 5′-Nucleotidase domain containing, OS = overall survival, PPS = post progression survival, RFS = relapse-free survival.

To evaluate prognostic value of the differentially expressed NT5DC family in breast cancer, we used the data from the gene chip in Kaplan–Meier Plotter to obtain survival analysis (Fig. 3). The results showed that all members had significant predictive value. The low expression level of NT5DC1 (*P* = 7.3e–06) and high expression level of NT5DC2 (*P* = 1.5e–06) indicated significantly shorter OS in breast cancer patients. and NT5C2, NT5DC3, and NT5DC4 expression levels did not correlate with the OS of these patients. Concerning DMFS, NT5C2 (*P* = 4.8e–04), NT5DC2 (*P* = 2.1e–09), and NT5DC3 (*P* = 1.5e–03) were correlated with poor DMFS, whereas NT5DC1 (*P* = 2.1e–04) and NT5DC4 (*P* = 3.1e–04) were correlated with long DMFS. As for PPS, poor PPS has been associated with high NT5C2 (*P* = 1.1e–05) and NT5DC2 (*P* = 2.1e–03) mRNA expression. Poor PPS was associated with low NT5DC1 mRNA expression (*P* = .025). However, NT5DC3 and NT5DC4 expression did not have a relationship with PPS.

Additionally, the prognostic value of the NT5DC family was examined via Bc-GenExMiner (see Figure S1, Supplemental Digital Content, http://links.lww.com/MD/I452, which illustrates the prognostic value of NT5DC family in breast cancer via bc-GenExMiner). We found that higher NT5C2, NT5DC2, and NT5DC4 mRNA expression had worse OS and DFS, while lower NT5DC1 and NT5DC3 mRNA expression had better OS and DFS.

We evaluated the effectiveness of NT5DC family expression levels in distinguishing breast cancer from normal tissues using ROC analysis (see Figure S2, Supplemental Digital Content, http://links.lww.com/MD/I453, which illustrates the diagnostic value of NT5DC family in breast cancer). The AUC values of NT5DC family members were all more than 0.6 (AUC = 0.662; 0.637; 0.623; 0.761; 0.837). The results revealed the diagnostic value of NT5DC family in breast cancer.

### 3.4. Genetic alterations, expression, and interactions of the NT5DC family in patients with breast cancer

**Figure 4.**
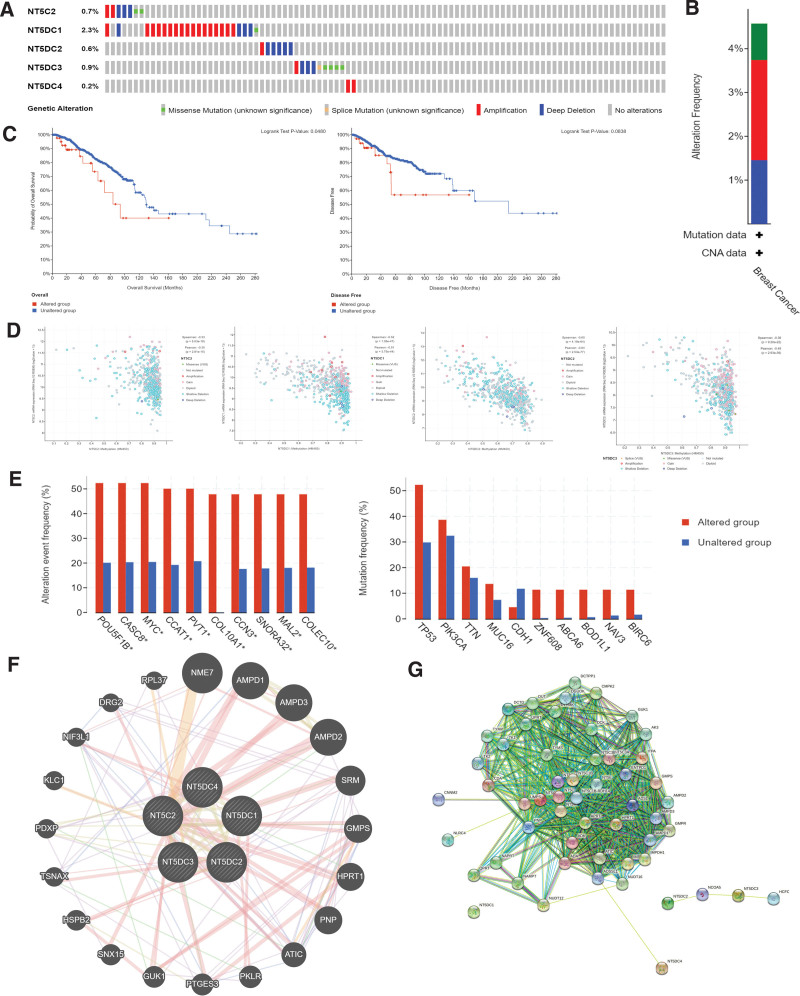
Genetic alteration, expression, and interaction analyses of NT5DC family among breast cancer patients. (A, B) The overview of alteration types and corresponding frequencies of NT5DC family in cBioPortal database. (C) The KM survival curves in different mutation status in cBioPortal database. (D) The relationship between DNA methylation and NT5DC family mRNA expression in cBioPortal database. (E) The relative gene alteration frequency in the altered and unaltered NT5DC groups in cBioPortal database. (F) Gene–gene interaction network of NT5DC family in GeneMANIA. (G) Protein-protein interaction network analysis of NT5DC family with STRING. NT5DC = 5′-Nucleotidase domain containing, STRING = Search Tool for the Retrieval of Interacting Genes.

Using the cBioPortal database, we explored the genetic alteration of the NT5DC family in breast cancer patients. In 963 breast cancer patients, genetic alternation was found in 44 patients, and the mutation rate was 5%. NT5C2, NT5DC1, NT5DC2, NT5DC3, and NT5DC4 were altered in 0.7%, 2.3%, 0.6%, 0.9%, and 0.2% (Fig. 4A). The alteration frequency was shown in Figure 4B. Kaplan–Meier plots and the Log-rank test were used to analyze the results, which indicated that genetic alterations in the NT5DC gene family were associated with poorer OS (Fig. 4C, *P* = .05). However, NT5DC family gene mutation did not significantly correlate with RFS (Fig. 4C, *P* = .08). The results of NT5DC family mRNA expression and DNA methylation were shown in Figure 4D, the expression of NT5DC families and DNA methylation except NT5DC4 (no data) showed a significant correlation. We also analyzed the relative gene alteration frequency in the altered and unaltered NT5DC groups. The most common gene mutations were TP53, PIK3CA, and TTN (Fig. 4E).

Furthermore, we used GeneMANIA to obtain potential interaction genes of the NT5DC family. Figure 4F shows the nodes surrounding five NT5DC genes, which are genes that are involved in physical interactions, co-expression, prediction, co-localization, genetic interactions, pathway, and shared protein domains. STRING was used to analyze the protein-protein interaction network of the NT5DC family (Fig. 4G). The network showed NT5DC family members and 50 proteins associated with them.

### 3.5. Functional enrichment analysis of NT5DC family in breast cancer

**Figure 5.**
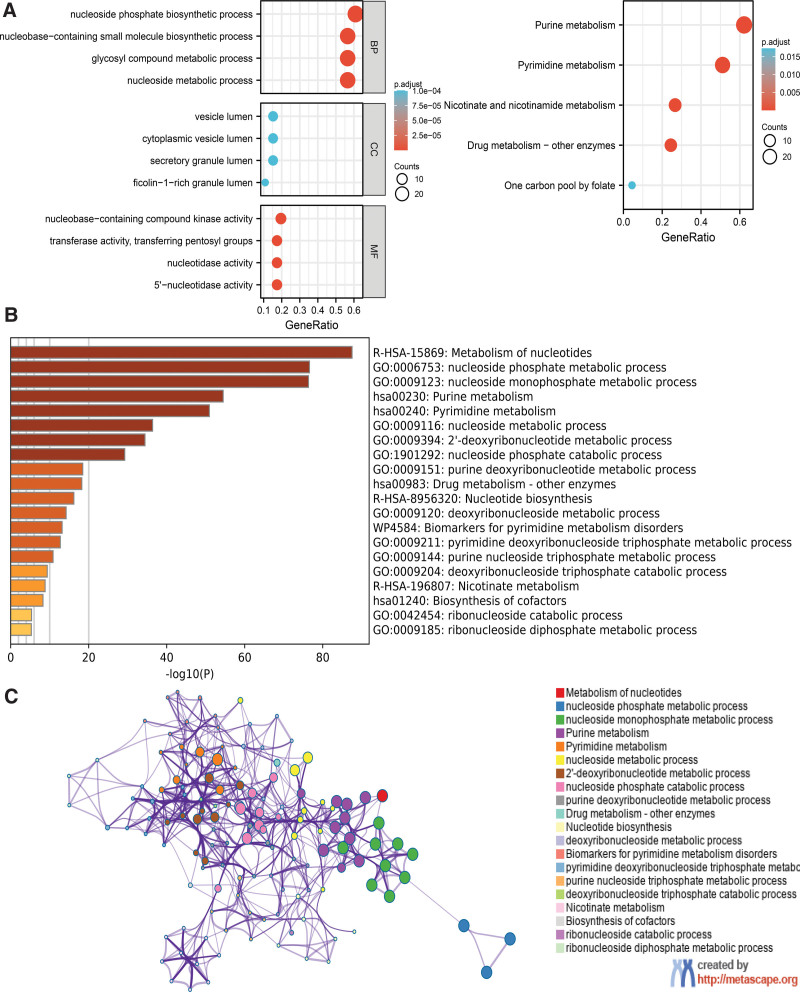
Functional and pathway enrichment analyses of NT5DC family. (A) GO and KEGG functional enrichment analyses. (B) The functional enrichment analysis in Metascape database. (C) Network of GO enriched terms colored by cluster ID in Metascape database. GO = gene ontology, KEGG =Kyoto Encyclopedia of Genes and Genomes, NT5DC = 5′-Nucleotidase domain containing.

GO and KEGG functional enrichment analyses were used to analyze NT5DC family and 50 neighbor genes (Fig. 5A). Most highly enriched GO items which found in the BP (biological processes) category were nucleoside phosphate biosynthetic process, nucleobase-containing small molecule biosynthetic process, glycosyl compound metabolic process and nucleoside metabolic process. In the CC (cellular components) category, vesicle lumen, cytoplasmic vesicle lumen, secretory granule lumen, and ficolin-1-rich granule lumen were related to the NT5DC family and their neighbor genes. In the MF (molecular functions) category, nucleobase-containing compound kinase activity, transferase activity, transferring pentosyl groups, nucleotidase activity, and 5′-nucleotidase activity were modulated by the NT5DC family and their neighbor genes in breast cancer. As for KEGG pathway analysis, purine metabolism, pyrimidine metabolism, nicotinate and nicotinamide metabolism, drug metabolism-other enzymes, and one carbon pool by folate were associated with the functions of the NT5DC family members and their neighbor genes in breast cancer.

Furthermore, we carried out functional enrichment analysis on the Metascape database. The functions of NT5DC genes and their neighboring genes were enriched in the metabolism of nucleotides, nucleoside phosphate metabolic process, nucleoside monophosphate metabolic process, purine metabolism and pyrimidine metabolism, as shown in Figure 5B. In Figure 5C, the network of enriched terms is color-coded according to the cluster ID.

### 3.6. Single-cell functional analysis of NT5DC family in breast cancer

**Figure 6.**
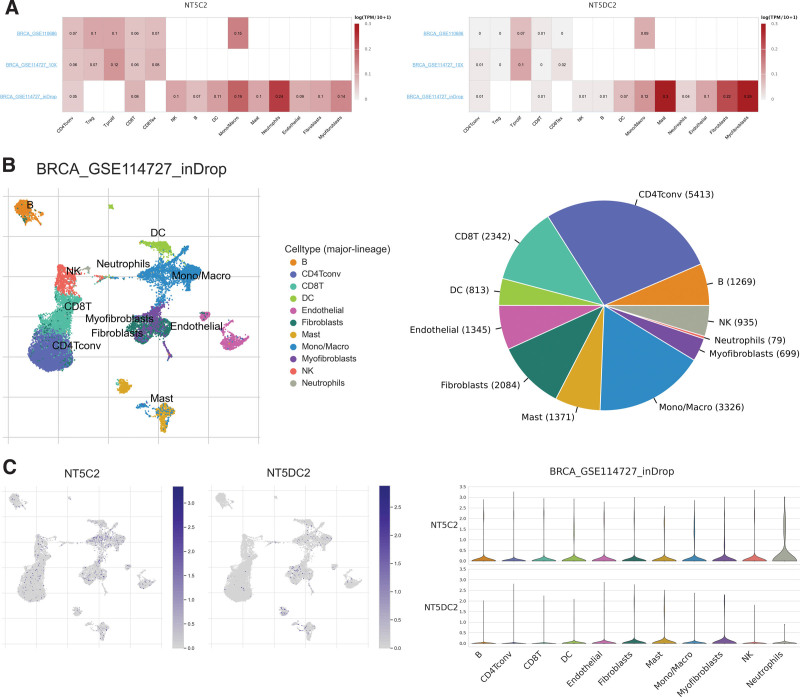
Single-cell level analysis of NT5DC family in TISCH. (A) Expression levels of NT5C2 and NT5DC2 in breast cancer microenvironment-associated cells in the GEO data set. (B) The cell types and their distribution in BRCA_GSE114727_inDrop dataset. (C) Distribution of NT5C2 and NT5DC2 in different cells in BRCA_GSE114727_inDrop dataset. BRCA = breast invasive carcinoma, NT5DC = 5′-Nucleotidase domain containing, TISCH = Tumor Immune Single-cell Hub.

Herein, we attempted to localize the NT5DC family at the single-cell level to evaluate their function. We used three datasets (BRCA_GSE110686, BRCA_GSE114727_10X, and BRCA_GSE114727_inDrop) of the TISCH web tool to interpret NT5C2 and NT5DC2 expression in tumor microenvironment-related immune cells. NT5C2 showed the highest degree of infiltration in Neutrophils, and NT5DC2 showed higher infiltration in Mast and Myofibroblasts (Fig. 6A). The figure indicates that NT5C2 is highly expressed in immune cells compared to NT5DC2. Then, the analysis was carried out on the BRCA_GSE114727_inDrop dataset, which comprised 11 types of cells (Fig. 6B). The pie chart showed that CD4 T cells accounted for the greatest proportion of this dataset. Figure 6C demonstrates that tumor microenvironment-related cells have a higher level of NT5C2 infiltration than NT5DC2, which is consistent with Figure 6A. At the single-cell level, these results suggest that NT5C2 and NT5DC2 play a vital role in the tumor microenvironment.

### 3.7. Immune infiltration analysis of NT5DC family in breast cancer

**Figure 7.**
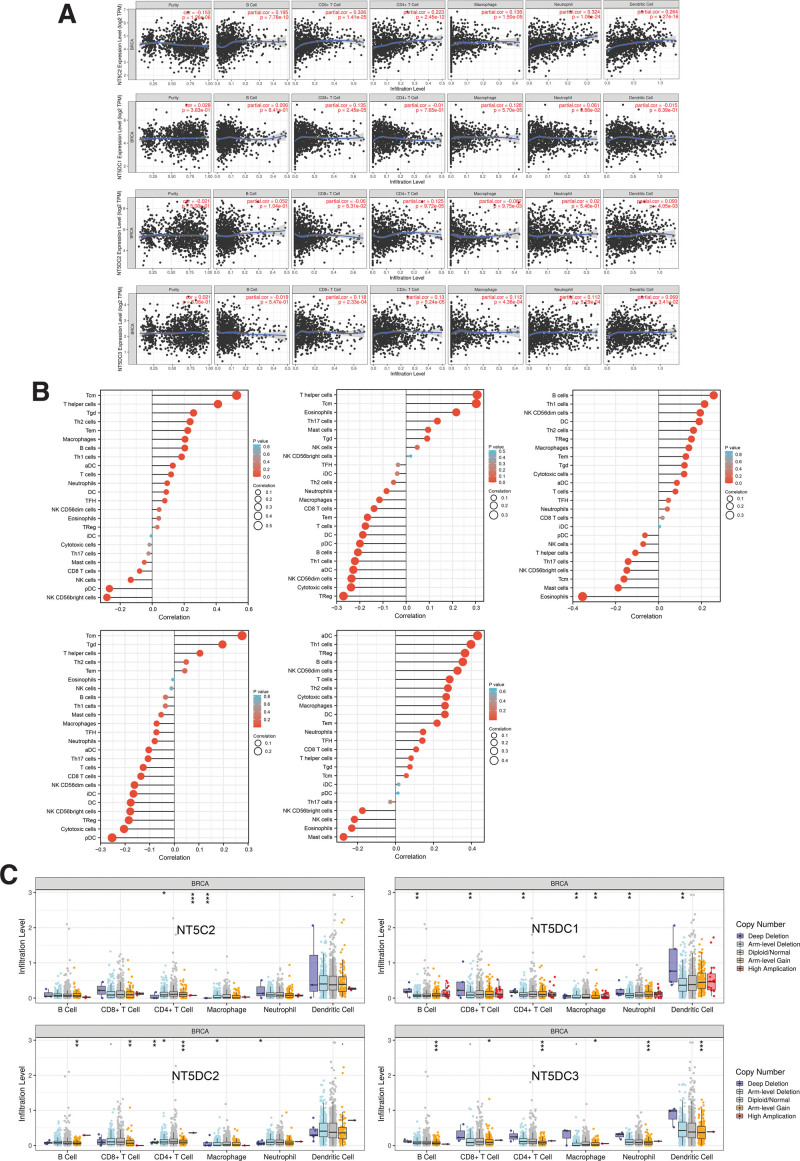
Relationship between NT5DC family expression and immune infiltration level in breast cancer. (A) The correlation between NT5DC family and immune cell infiltration (TIMER). (B) Association of NT5DC family expression with the infiltration of immune cells (ssGSEA). (C) The relationship of the SCNA of NT5DC family and the infiltration levels of six immune cells. *P < .05, **P < .01, ***P < .001. BRCA = breast invasive carcinoma, NT5DC = 5′-Nucleotidase domain containing, ssGSEA = single sample gene set enrichment analysis, TIMER = Tumor Immune Estimation Resource.

Utilizing the TIMER database, we examined the correlation between NT5DC family and immune cell infiltration (Fig. 7A). NT5C2 expression was positively connected with the infiltration of all 6 types of immune cells. Infiltration of CD8+T cells (*P* = 2.45e–05) and macrophages (*P* = 5.7e–05) was positively correlated with NT5DC1 expression. As for NT5DC2, it was positively associated with CD4+T cells infiltration (*P* = 9.72e–05), dendritic cells infiltration (*P* = 4.05e–03), and negatively associated with macrophages infiltration (*P* = 9.75e–03). The levels of NT5DC3 and infiltration of CD8+T cells (*P* = 2.33e–04), CD4+T cells (*P* = 5.24e–05), macrophages (*P* = 4.36e–04), neutrophils (*P* = 5.29e–04), and dendritic cells (*P* = 3.41e–02) were positively correlated. Additional, ssGSEA analysis showed that NT5DC family expression had a significant correlation with 24 immune cells (Fig. 7B).

Furthermore, we investigated the somatic copy number alteration (SCNA) of the NT5DC family in breast cancer (Fig. 7C). NT5DC1 was significantly associated with arm-level deletion of 6 immune cells, and NT5DC3 was associated with arm-level gain of six immune cells. NT5C2 showed a significant correlation with arm-level deletion and high amplification of CD4+T cell and deep deletion of macrophages. NT5DC2 showed a significant correlation with CNA levels of all immune cells except for dendritic cells.

### 3.8. Relationship between NT5DC family expression and immune subtypes, tumor immune microenvironment and immune checkpoints

**Figure 8.**
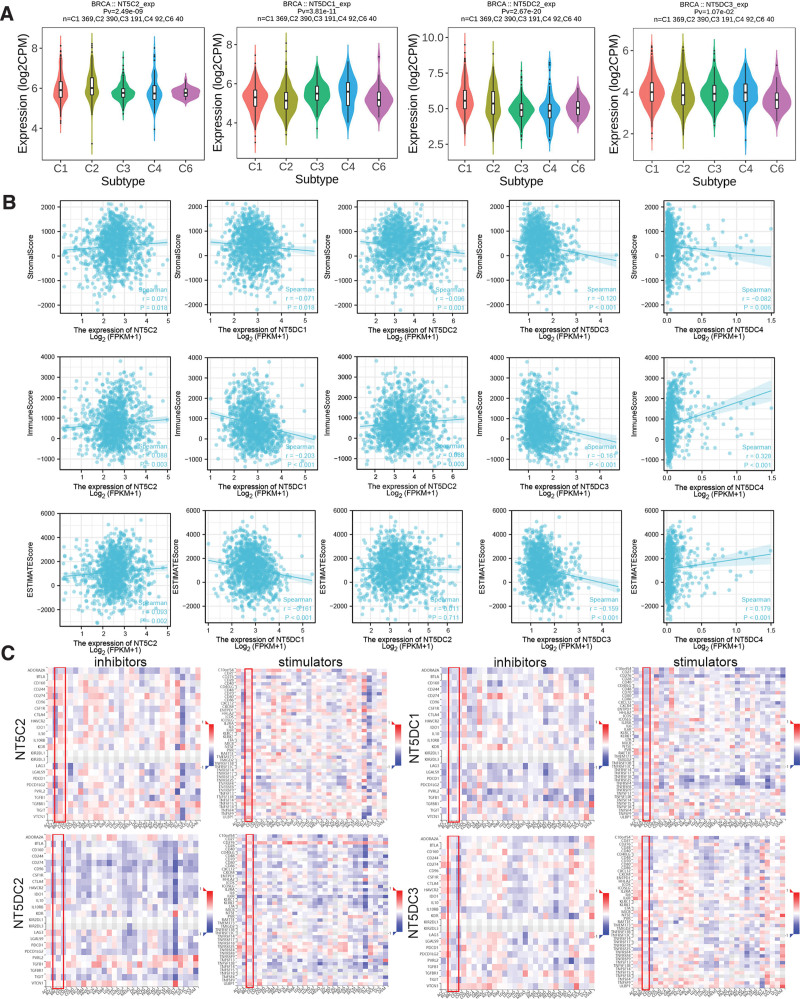
Relationship between NT5DC family expression and immunity. (A) Violin plots showed the relationship between NT5DC family and immune subtypes of breast cancer. (B) Scatter plots showed the association between NT5DC family and tumor immune microenvironment. (C) Heatmaps showed the relationship between NT5DC family and immunecheckpoint related genes. BRCA = breast invasive carcinoma, NT5DC = 5′-Nucleotidase domain containing.

Based on the TISIDB database, we examined the association between the NT5DC family and immune subtypes (C1 wound healing, C2 IFN-γ dominant, C3 inflammatory, C4 lymphocyte depleted, C5 immunologically quiet, C6 TGF-β dominant) (Fig. 8A). NT5DC family members were all significantly related to different immune subtypes. As shown in the figure, a higher level of NT5C2 and NT5DC1 expression was observed in C1-4 and C6. NT5DC2 expression was higher in C1 and lower in C3. The expression of NT5DC3 was related to C1 subtype.

In terms of Stromal Score, NT5C2 (*P* = .02) showed positive correlation, while NT5DC1 (*P* = .02), NT5DC2 (*P* = .001), NT5DC3 (*P* < .001), and NT5DC4 (*P* = .006) showed negative correlation. The expression of NT5C2 (*P* = .003), NT5DC2 (*P* = .003), and NT5DC4 (*P* < .001) was positively associated with Immune Score, while the expression of NT5DC1 (*P* < .001) and NT5DC3 (*P* < .001) was negatively associated with Immune Score. As for ESTIMATE Score, NT5C2 (*P* = .002) and NT5DC4 (*P* < .001) had positive correlation, and NT5DC1 (*P* < .001) and NT5DC3 (*P* < .001) had negative correlation. Based on the above results, a significant correlation has been found between NT5DC family expression and immune scores in breast cancer in tumor immune microenvironment (Fig. 8B).

Inhibitory and stimulatory immune checkpoints regulate immune escape and immune efficacy. By using the TISIDB database, we determined where NT5DCs are expressed in relation to 69 immune checkpoint genes (24 inhibitors and 45 stimulators) (Fig. 8C). We found that NT5C2 expression was significantly related to 16 immune checkpoint inhibitors and 32 immune checkpoint stimulators (see Table S1, Supplemental Digital Content, http://links.lww.com/MD/I454, which illustrates significant relationship between NT5C2 expression and immune checkpoint related genes). The expression of NT5DC1 was significantly associated with 17 immune checkpoint inhibitors and 33 immune checkpoint stimulators (see Table S2, Supplemental Digital Content, http://links.lww.com/MD/I455, which illustrates significant relationship between NT5DC1 expression and immune checkpoint related genes). NT5DC2 expression was significantly related to 8 immune checkpoint inhibitors and 26 immune checkpoint stimulators (see Table S3, Supplemental Digital Content, http://links.lww.com/MD/I456, which illustrates significant relationship between NT5DC2 expression and immune checkpoint related genes). The expression of NT5DC3 was significantly associated with 17 immune checkpoint inhibitors and 24 immune checkpoint stimulators (see Table S4, Supplemental Digital Content, http://links.lww.com/MD/I457, which illustrates significant relationship between NT5DC3 expression and immune checkpoint related genes).

### 3.9. Drug sensitivity analysis

**Figure 9.**
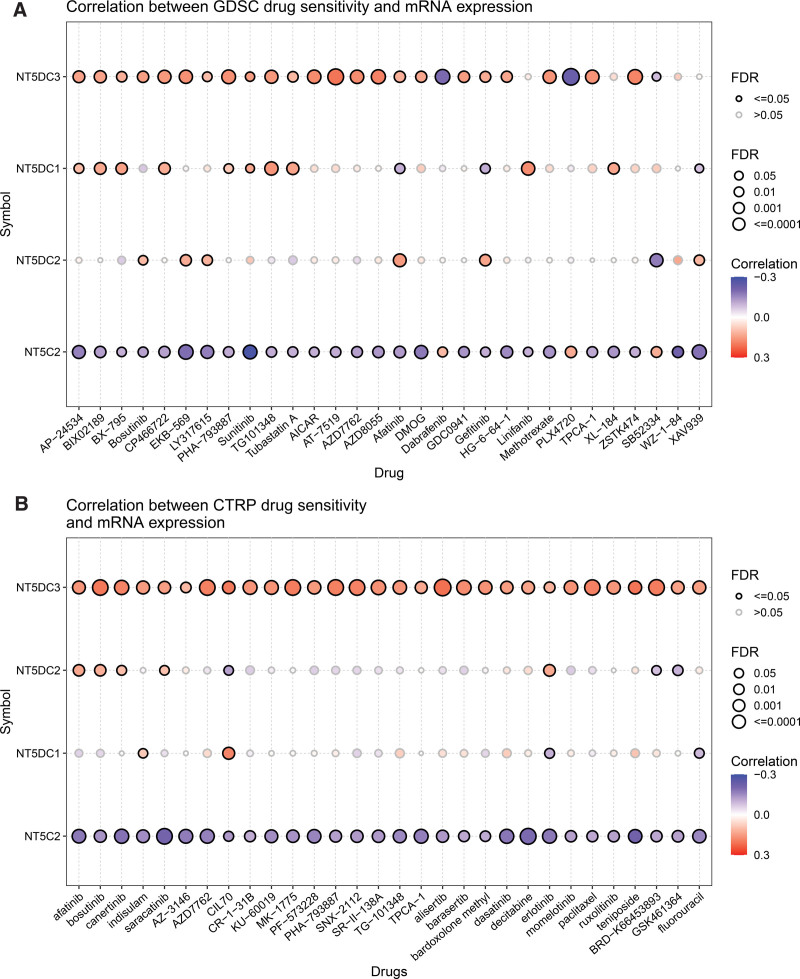
Correlations between NT5DC family and drug sensitivity from GSCALite database. (A) Bubble plot showed the correlation between the expression of NT5DC family and the sensitivity of GDSC drugs (top 30) in pan-cancer. (B) The correlation between the expression of NT5DC family and the sensitivity of CTRP drugs (top 30) in pan-cancer. CTRP = Cancer Therapeutics Response Portal, GDSC = Genomics of Drug Sensitivity in Cancer, GSCALite = Gene Set Cancer Analysis, NT5DC = 5′-Nucleotidase domain containing.

To reveal the responsiveness of NT5DC family to chemotherapy, we explored the associations between the expression of NT5DCs and drug sensitivity through GDSC and CTRP databases. In GDSC database, low NT5C2 expression was resistant to most of drugs or small molecules such as AP−24534, BIX02189, BX-795, Bosutinib, CP466722, EKB−569, LY317615, PHA−793887, Sunitinib, TG101348, Tubastatin A, AICAR, AT−7519, AZD7762, AZD8055, Afatinib, DMOG, GDC0941, Gefitinib, HG−6−64−1, Linifanib, Methotrexate, TPCA−1, XL−184, ZSTK474, WZ−1−84 and XAV939, whereas high expression of NT5DC3 was resistant to these drugs (Fig. 9A). Based on the CTRP database, NT5C2 was negatively correlated to the IC50 of fatinib, bosutinib, canertinib, indisulam, saracatinib, AZ−3146, AZD7762, CIL70, CR−1−31B, KU−60019, MK−1775, PF−573228, PHA−793887, SNX−2112, SR−II−138A, TG−101348, TPCA−1, alisertib, barasertib, bardoxolone methyl, dasatinib, decitabine, erlotinib, momelotinib, paclitaxel, ruxolitinib, teniposide, BRD−K66453893, GSK461364 and fluorouracil, while NT5DC3 was positively correlated to the IC50 of these drugs (Fig. 9B).

## 4. Discussion

The most frequent malignancy in women is breast cancer,^[33]^ which is a molecularly heterogeneous illness.^[34]^ Although diagnosis and treatment of breast cancer have improved, the prognosis of patients with breast cancer is still poor.^[4]^ Therefore, exploring new meaningful biomarkers for diagnosis and prognosis is required and urgent. NT5DC family is evolutionary conserved 5′-nucleotidase enzymes that can catalyze the hydrolysis of nucleotides in cells and comprise five members (NT5C2, NT5DC1, NT5DC2, NT5DC3, and NT5DC4).^[5]^ In recent years, cancer susceptibility and development are associated with NT5DC family members. The expression of NT5C2 plays an important role in lung cancer,^[10]^ acute leukemia,^[35]^ and astrocytoma.^[36]^ In breast cancer, by regulating ROS metabolism and defense, NT5C2 regulates cellular responses to glucose deprivation.^[19]^ The expression of NT5DC2 was correlated with glioblastoma,^[13]^ hepatocellular carcinoma,^[15]^ colorectal carcinoma,^[17]^ lung cancer,^[37]^ and leiomyosarcoma.^[38]^ There are no studies about NT5DC1, NT5DC3, and NT5DC4 in cancer. Previous studies almost focused on single NT5DC family member and single aspect. There is no study exploring the comprehensive analysis of the NT5DC family in breast cancer at present.

In this study, the aspects of expression, prognostic value, gene alteration, function and immune infiltration of the NT5DC family in breast cancer are analyzed comprehensively for the first time. According to our study, the expression of NT5C2, NT5DC1, and NT5DC3 was downregulated, while the expression of NT5DC2 was upregulated in breast cancer tissues. All NT5DC family members was associated with clinicopathological parameters of breast cancer patients. Survival analysis showed that high expression of NT5C2 had poor RFS, DMFS, and PPF in patients with breast cancer. Poor OS, RFS, DMFS, and PPS were associated with low NT5DC1 expression and high NT5DC2 expression. NT5DC3 and NT5DC4 expression was related to RFS and DMFS. The ROC analysis revealed the diagnostic value of NT5DC family. These results revealed that NT5DC family members were significantly associated with the prognosis and diagnosis of breast cancer patients.

We also explored the genetic alteration of NT5DC family in breast cancer via the cBioPortal database. The results showed that 5% of the breast cancer cases had NT5DC family gene alterations. Moreover, the altered NT5DC group had worse OS compared with the unaltered NT5DC group. As for DNA methylation, all NT5DC family members were significantly associated with it, suggesting that epigenetic regulation participated in NT5DC family-induced biological properties of breast cancer. We also analyzed the relative gene alteration frequency in the altered and unaltered NT5DC groups. The most common gene mutations were TP53, PIK3CA, and TTN. In the altered NT5DC family group, these genes had higher mutation frequency. Previous studies showed that mutated TP53 and PIK3CA could be used as biomarkers and therapeutic targets.^[39,40]^ A TTN biomarker could also be used to diagnose and prognosis breast cancer.^[41]^

We then explored the genes that are related to NT5DC family, such as NME7, AMPD1, and AMPD2. Previous studies have demonstrated the relationship of them and breast cancer. NME7 might play tumor-suppressive role in breast cancer.^[42]^ In HER2+breast cancer, AMPD1 might be a promising biomarker to predict disease outcomes.^[43]^ Adedokun et al^[44]^ also found that one of the breast cancer loci is near the AMPD2 gene. We further carried out functional enrichment analysis. The results showed that NT5DC family and its related genes were mainly correlated with the metabolism of nucleotides. There are reports showed that NT5DC family may be associated with nucleotide metabolism.^[5,6]^ Moreover, previous studies have explored that nucleotide metabolism contributes to cancer initiation and progression.^[45–47]^ Therefore, NT5DC family may regulate the occurrence and development of breast cancer through suppressing the nucleotide metabolism pathway.

Tumor microenvironment consists of tumor-associated endothelial cells, immune cells, cancer-associated fibroblasts, vasculature, and extracellular matrix.^[48]^ In addition to playing a key role during tumor initiation, progression, and metastasis, the tumor microenvironment also influences the therapeutic response.^[49,50]^ In our study, we conducted single-cell level analyses via TISCH database. Breast cancer contains multiple cell types, which includes B, NK, CD4 T conv, CD8 T, Neutrophils, Mast, Endothelial, DC, Fibroblasts, Mono/Macro, and Myofibroblasts. NT5DC family members were expressed in major immune cells to different degrees. NT5C2 was mainly expressed in Neutrophils, whereas NT5DC2 was mainly expressed in Mast and Myofibroblasts.

It has been suggested that tumor-infiltrating lymphocytes (TILs) have the potential to affect tumor progression, recurrence, and to predict the prognosis of cancer patients as well as the effectiveness of immunotherapy.^[51–53]^ There was a strong association between the NT5DC family and the six most common types of immune cell infiltration in our study. All members of the NT5DC family were significantly related to different immune subtypes as well. Moreover, NT5DC family was correlated with some well-known immune checkpoint inhibitors and stimulators. These results proved the potential immune function of NT5DC family in breast cancer. We then explored the tumor immune microenvironment. A negative correlation was observed between NT5DC1 and NT5DC3 and stromal and immune scores. NT5C2 was positively associated with stromal and immune scores. Low stromal score and high immune score were associated with high expression of NT5DC2 and NT5DC4. These results suggested that NT5DC family might be potential targets for breast cancer immunotherapy.

Metastatic breast cancer is still incurable and the therapeutic options are limited.^[54]^ Drug sensitivity analysis showed that NT5C2 was negatively correlated to the IC50 of most drugs and NT5DC3 was positively correlated to the IC50 of most drugs. These results suggested that NT5C2 and NT5DC3 are potential biomarkers for drug screening. Immunotherapy is becoming the most promising strategy for cancer treatment, and immune checkpoint blockade is major part of immunotherapy.^[55]^ We also revealed that NT5DC family members were strongly associated with immune checkpoints and played vital roles in tumor immune microenvironment. Therefore, the combination of immunotherapy and chemotherapy targeting NT5DC family could provide a novel therapeutic option for breast cancer.

Limitations are existed in present study. First, we analyzed the NT5DC family by using online datasets, and the data and clinical information were limited. In addition, our study only explored the bioinformatical associations between NT5DC family and breast cancer. The detailed mechanisms of NT5DC family on breast cancer needs further studies.

## 5. Conclusion

In this study, the expression, prognosis, diagnosis, function and immune significance of NT5DC family were comprehensively analyzed in breast cancer, indicating that NT5DC family members may serve as new prognostic biomarkers and potential immunotherapeutic targets. Our study provides new findings of NT5DC family and NT5DC family members’ mechanisms of action and their effectiveness in treating breast cancer patients need to be further investigated.

## Acknowledgments

We appreciated a lot for the public databases and The Cancer Genome Atlas (https://portal.gdc.cancer.gov/).

## Author contributions

**Conceptualization:** Yiwei Jia, Fei Wu.

**Data curation:** Yiwei Jia, Jia Li, Huizi Wu.

**Formal analysis:** Yiwei Jia, Weiwei Wang, Shiyu Sun.

**Funding acquisition:** Fei Wu.

**Methodology:** Yiwei Jia, Cong Feng, Xuan Liu.

**Project administration:** Yiwei Jia, Yu Zhang.

**Software:** Yiwei Jia, Chaofan Li, Yifan Cai.

**Supervision:** Fei Wu, Shuqun Zhang.

**Validation:** Yiwei Jia, Xuanyu Liu, Xinyu Wei.

**Visualization:** Yiwei Jia, Peizhuo Yao.

**Writing – original draft:** Yiwei Jia.

**Writing – review & editing:** Fei Wu, Shuqun Zhang.

## Supplementary Material












