# Supplementary material for: Comprehensive analysis of NT5DC family prognostic and immune significance in breast cancer
Source: Medicine (Baltimore). 2023 Feb 10;102(6):e32927. doi: 10.1097/MD.0000000000032927 (PMC9907984; doi:10.1097/MD.0000000000032927)

Figure S1. The prognostic value of NT5DC family in breast cancer via bc-GenExMiner. The survival curves of NT5C2 (A), NT5DC1 (B), NT5DC2 (C), NT5DC3 (D), and NT5DC4 (E). OS, overall survival; DFS, disease-free survival.

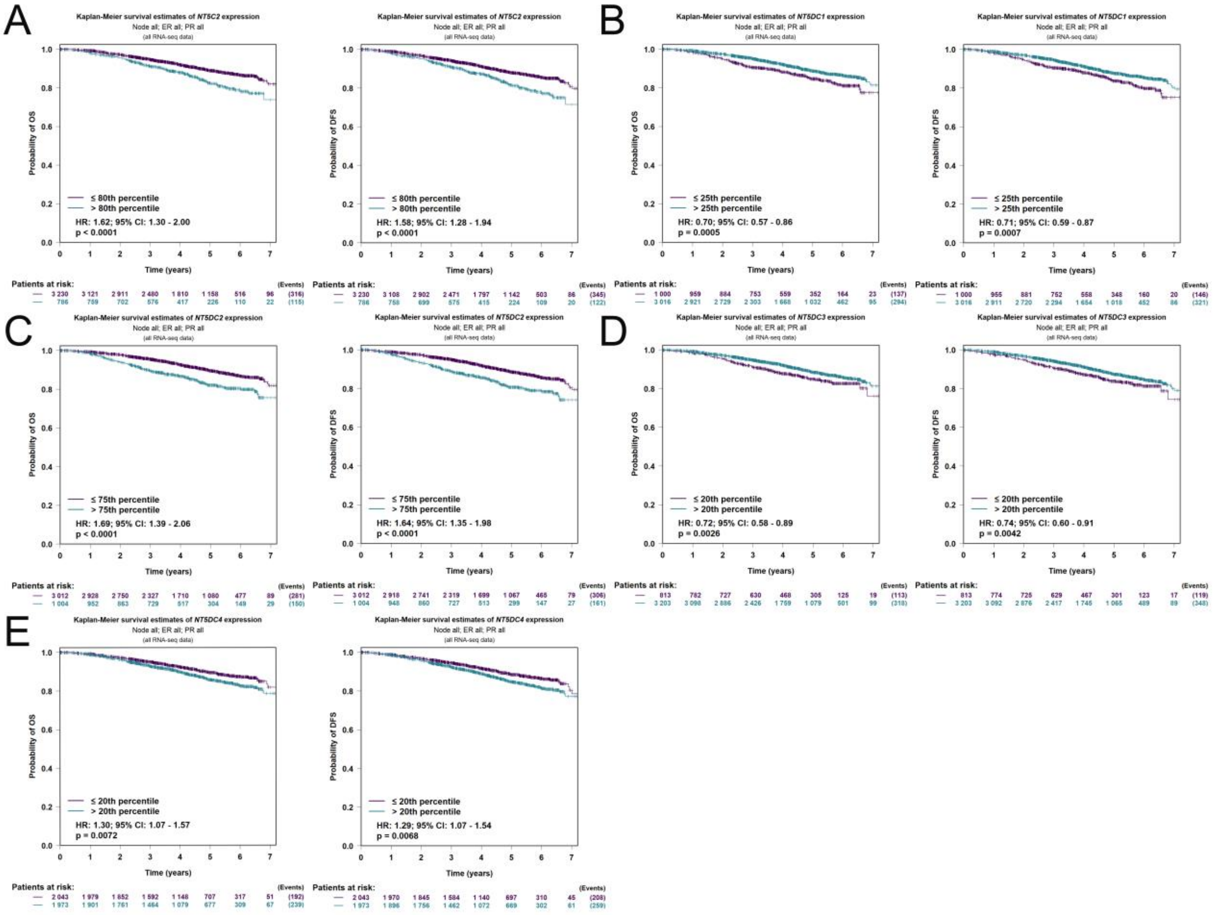

Supplement: Supplementary file 1 [file medi-102-e32927-s001.pdf]
