# Supplementary material for: Comprehensive analysis of NT5DC family prognostic and immune significance in breast cancer
Source: Medicine (Baltimore). 2023 Feb 10;102(6):e32927. doi: 10.1097/MD.0000000000032927 (PMC9907984; doi:10.1097/MD.0000000000032927)

Figure S2. The diagnostic value of NT5DC family in breast cancer. The ROC curve of NT5C2 (A), NT5DC1 (B), NT5DC2 (C), NT5DC3 (D) and NT5DC4 (E).

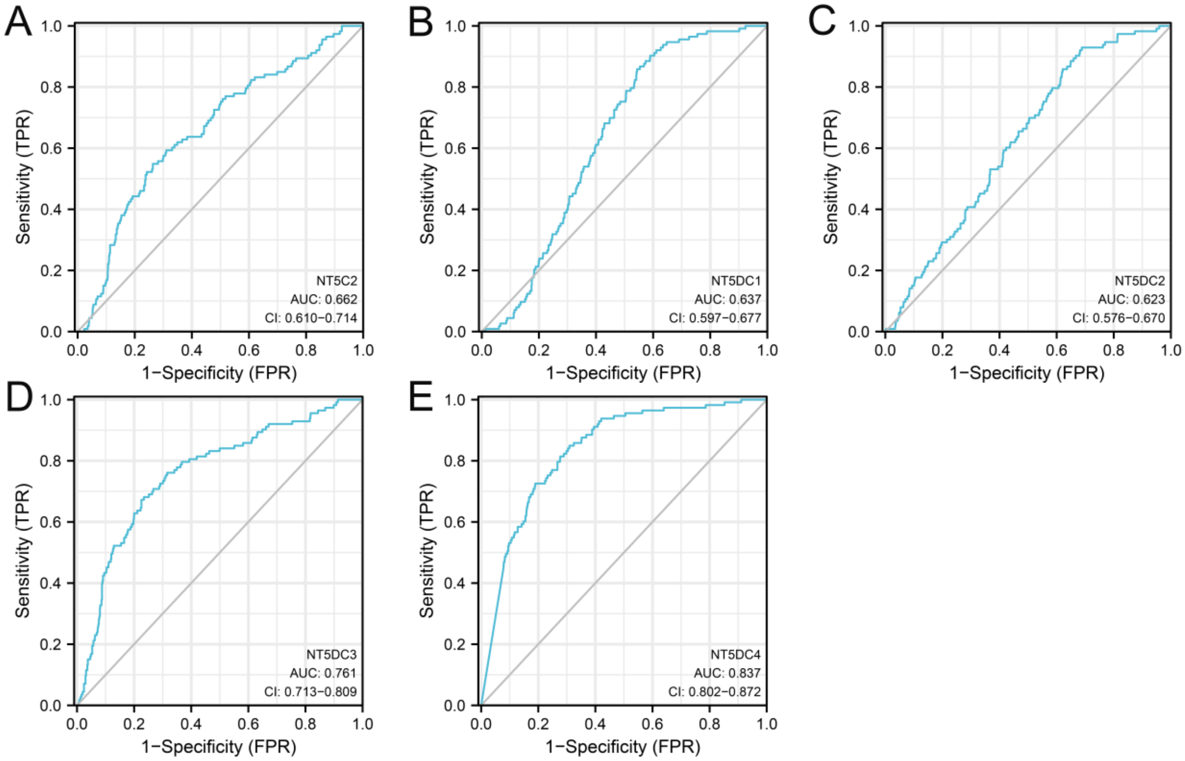

Supplement: Supplementary file 2 [file medi-102-e32927-s002.pdf]
